# Promoting vaccination in maternity wards ─ motivational interview technique reduces hesitancy and enhances intention to vaccinate, results from a multicentre non-controlled pre- and post-intervention RCT-nested study, Quebec, March 2014 to February 2015

**DOI:** 10.2807/1560-7917.ES.2019.24.36.1800641

**Published:** 2019-09-05

**Authors:** Arnaud Gagneur, Marie-Claude Battista, François D. Boucher, Bruce Tapiero, Caroline Quach, Philippe De Wals, Thomas Lemaitre, Anne Farrands, Nicole Boulianne, Chantal Sauvageau, Manale Ouakki, Virginie Gosselin, Geneviève Petit, Marie-Claude Jacques, Ève Dubé

**Affiliations:** 1 Centre de recherche du CHUS, Sherbrooke, Québec, Canada; 2 Centre hospitalier universitaire de Sherbrooke, Université de Sherbrooke, Sherbrooke, Québec, Canada; 3 Department of Medicine, Faculty of Medicine and Health Sciences, Université de Sherbrooke, Sherbrooke, Québec, Canada; 4 Centre de recherche du Centre Hospitalier Universitaire de Québec, Québec, Québec, Canada; 5 CHU Sainte Justine, Université de Montréal, Montréal, Québec, Canada; 6 McGill University Health Centre Research Institute – Vaccine Study Centre, Montréal, Québec, Canada; 7 Department of Social and Preventive Medicine, Laval University, Québec, Québec, Canada; 8 Institut national de santé publique du Québec, Québec, Québec, Canada; 9 Direction de santé publique du CIUSSS de l’Estrie – CHUS, Département des sciences de la santé communautaire, Université de Sherbrooke, Sherbrooke, Québec, Canada; 10 Institut universitaire de première ligne en santé et services sociaux du CIUSSS de l’Estrie – CHUS, Québec, Canada

**Keywords:** vaccine, hesitancy, intention, motivational interviewing, infant, parents, Canada, vaccines, immunisation

## Abstract

**Background:**

Many countries are grappling with growing numbers of parents who delay or refuse recommended vaccinations for their children. This has created a need for strategies to address vaccine hesitancy (VH) and better support parental decision-making regarding vaccination.

**Aim:**

To assess vaccination intention (VI) and VH among parents who received an individual motivational-interview (MI) based intervention on infant immunisation during post-partum stay at a maternity ward between March 2014 and February 2015.

**Methods:**

This non-controlled pre-/post-intervention study was conducted using the results from parents enrolled in the intervention arm of the PromoVaQ randomised control trial (RCT), which was conducted in four maternity wards across the Province of Quebec. Participants (n = 1,223) completed pre- and post-intervention questionnaires on VI and VH using Opel’s score. Pre-/post-intervention measures were compared using McNemar’s test for categorical variables and Wilcoxon signed-rank test for continuous variables.

**Results:**

Pre-intervention: overall VI was 78% and significantly differed across maternity wards (74%, 77%, 84%, 79%, p = 0.02). Post-intervention: VI rose significantly across maternity wards (89%, 85%, 95%, 93%) and the overall increase in VI was 12% (78% vs 90%, p < 0.0001). VH corroborated these observations, pre- vs post-intervention, for each maternity ward (28% vs 16%, 29% vs 21%, 27% vs 17%, 24% vs 13%). Overall, VH was curbed post-intervention by 40% (27% vs 16%; p < 0.0001).

**Conclusions:**

Compared with pre-intervention status, participants who received the MI-based intervention on immunisation displayed lower hesitancy and greater intention to vaccinate their infant at 2 months of age.

## Introduction

According to data from the World Health Organization (WHO), 19.5 million children worldwide failed to receive routine life-saving vaccinations in 2016 while ca 90,000 children died from measles, a vaccine-preventable disease [1]. These figures suggest that vaccination, long recognised as instrumental to human health, still faces complex and multi-factorial barriers leading many families to forego or delay childhood immunisation [2]. Despite past and ongoing campaigns to promote childhood vaccination, including efforts to facilitate vaccination, current worldwide vaccine coverage against diphtheria-tetanus-pertussis (DTP3) is ca 85%, which is less than the expected threshold of 90% for herd immunity [3]. In the Province of Quebec (Canada), the latest survey conducted by the National Institute of Public Health of Quebec showed that, as of 2016, complete vaccine coverage (including against rotavirus and hepatitis B) was reached for 82% of children aged 24 months [4]. The Quebec immunisation schedule can be seen in Supplement S1. Only 50% of children aged 24 months received all recommended vaccinations (excluding rotavirus and hepatitis B) within 1 month after the recommended age for each dose [4].

A reason for falling vaccine coverage is parental vaccine hesitancy (VH); a concept first recognised by the WHO Strategic Advisory Group of Experts (SAGE) on Immunisation in 2012, with a clear definition published in 2015 [5]. In response to this definition, an online survey was conducted among Canadian parents to explore the degree/level of VH in Canada in 2015 by the Canadian Immunization Research Network. A total of 2,013 parents/caregivers of at least one child (aged 24–59 months) participated. They reported that 85% of the children under their care had received all of the recommended vaccines according to the schedule [6] and there was an overall positive attitude towards immunisation. Further, the levels of parental vaccination awareness and trust in institutions associated with VI was positive [6]. In the Province of Quebec, higher VH was associated with low household income and low education level [7].

Face-to-face interventions have been proposed as a strategy to address VH and to increase vaccination awareness among parents. A scoping review and meta-analysis, published in 2015, concluded that while there is no strong evidence to support the use of any specific intervention to address VH [8], interventions directly tailored at vaccine-hesitant parents were scarce. In 2018, a Cochrane Review concluded that low to moderate evidence suggested that face-to-face interventions might improve parental VI if adapted to the target population and provide accurate information on vaccines [9].

Traditional educational methods (e.g. information pamphlets, communication interventions aiming to provide information) have proven inefficient in addressing VH [10]. It is known that merely providing additional factual information to vaccine-hesitant parents is counterproductive [11]. Our group developed a vaccination promotion programme, called PromoVac, based on a face-to-face intervention with parents conducted post-partum in maternity wards. We further refined the intervention using a standardised information session and motivational-interview (MI) techniques [12,13]. Our novel face-to-face intervention strategy is patient-oriented, tailored to welcome parents at their individual level of knowledge and with respectful acceptance of their personal beliefs [14]. Our first quasi-experimental regional pilot study (‘PromoVac’) using this MI-based intervention was conducted in the Eastern Townships region of the Province of Quebec between March 2010 and February 2011. Locally, results demonstrated both an increase in parents’ VI (15%) and in the vaccine coverage (7%) of infants aged 7 months [12,13], suggesting potential benefits. Results on the long-term impact of our MI-based post-partum intervention show that the children of participant parents who received it were 9% more likely to display complete vaccine coverage at 0–2 years [15].

The ‘PromoVaQ’ study aimed to scale-up our regional pilot, monocentric, quasi-experimental study (‘PromoVac’ March 2010–February 2011) to a Province-wide multicentric study, conducted in four university hospital maternity wards between March 2014 and February 2015, in order to measure how our MI-based post-partum intervention impacted post-intervention VI and VH in participant parents of newborns.

## Methods

### Design

To assess the post-intervention impact on VI and VH, we designed a nested non-controlled pre-/post-intervention study using data from consenting parents enrolled in the intervention arm of a pragmatic, unblinded, parallel-randomised controlled trial (RCT) (NCT02666872); this study design is recognised as being suitable to determine the impact of an experimental intervention in a single arm study [16].

This study was a pragmatic, unblinded, parallel-randomised controlled trial (RCT) powered to compare the impact of our MI-based intervention to the standard of care provided to parents of 2-day-old newborns on the overall vaccine coverage for children aged 24 months (refer to the study protocol for additional details [17]).

### Setting

The RCT was conducted in four university hospital maternity wards of the Province of Quebec, collectively accounting for over 20% of all births province wide. The hospitals were located in Sherbrooke (CIUSSS de l’Estrie - CHUS), Montreal (in a French- and an English-language maternity ward at the CHU Ste-Justine Hospital and the McGill University Health Centre, respectively) and Québec city (CHU de Quebec). These hospitals were selected in order to increase external generalisability of results, characterise feasibility issues and determine efficacy of the intervention, irrespective of regional disparities in maternity ward organisation and/or socioeconomic and cultural diversities. However, it was beyond the scope of this study to further dissect sites differences.

### Study period, population and eligibility criteria

Enrolment took place between March 2014 and February 2015. Mothers were eligible to participate in the study if their newborn was delivered in one of the four participating university hospital maternity wards and they had not yet been discharged. Mothers were excluded if: (i) they were aged 18 years or younger, (ii) did not speak either French or English, (iii) participated in the pilot study conducted at the CIUSSS de l’Estrie - CHUS between 2010 and 2011, (iv) if their newborn presented an unstable condition requiring intensive care management, or (iv) if interviewing was incompatible with the mother’s health. If the father was also at the maternity ward, he was invited to receive the intervention and answer the questionnaires jointly with the mother.

Parents who consented to participate in the study were randomised through a web-based system (Dacima). Randomisation was conducted using a block size strategy (eight participants/block) and was stratified by maternity ward using a 1:1 allocation ratio to ensure proportionate allocation among sites and groups.

Parents enrolled on the standard of care arm of the RCT did not complete post-intervention questionnaires, as it has been shown that providing parents with a copy of the public health vaccine brochure (standard of care), does not alter parental VI or VH [18].

### Ethics

This study was reviewed and approved by the institutional research ethics review board at each site ((CIUSSS de l’Estrie – CHUS: 2014-609, 13-074; McGill University Health Centre: 13-084 (3262); CHU Ste-Justine: 2014-601, 3793; CHU de Québec: 2014-1742, B13-07-1742)). Written informed consent was obtained from all participants before study inclusion and participation as required by law.

### Intervention

The study intervention has been described previously [12,17]. Briefly, the intervention merges the MI framework [14] to Prochaska’s stages-of-change model as the conceptual backbone [19]. According to this model, stepwise changes [19] must occur in order to increase an individual’s awareness and internal motivation to change by exploring/resolving his/her own ambivalences [14]. The rationale underlying the study intervention was to accompany parents, in a non-judgmental manner, from their own stage of VI to the next stage by tailoring the intervention accordingly. The intervention covered five main areas: (i) vaccine-preventable diseases and their consequences, (ii) vaccines and their effectiveness, (iii) the importance of the immunisation calendar in infants, (iv) reluctance to vaccinate and vaccination side-effects [20], and (v) vaccination services and facilities in each of the study regions. Local research assistants were trained to provide a standardised intervention and a 2-week trial period was conducted at each maternity ward before the study launch. The MI-based intervention was administered individually to consenting parents 24–48 hours after delivery in their maternity ward room. The intervention lasted ca 20 min. Based on the pragmatic nature of this RCT, co-interventions were allowed and maternity staff interacted with the participants based on their clinical judgement.

### Outcomes and measurement tools

The primary outcome was VI measured using a validated questionnaire [12,17,21] based on the health belief model [22], where answers were provided according to a four-category Likert scale (certainly not, probably not, probably and certainly). The secondary outcome was parental VH measured using Opel’s validated questionnaire [12]. Briefly, VH questions were scored in an adapted Opel approach [23] as follows: 2 points for hesitant-related responses; 1 point for ‘I don’t know or not sure’ responses and 0 for non-hesitant responses. Scores were summed unweighted to a 0–100 range using simple linear transformation and accounting for missing data. According to the methodology of Opel [23] and Dube [24], categories were defined as follows: 0–29 score = low level VH; 30–49 = moderate level VH; 50 and higher = high level VH. Questionnaires were self-administered and distributed to parents before and immediately following the end of the MI-based intervention. The post-intervention questionnaire was collected at discharge from the maternity ward.

### Statistical analyses

As this study is nested within a larger RCT’s objective, no sample-size calculation was defined a priori to answer this study’s primary outcome. Based on our previous study evaluating a 77.5% baseline VI in parents [24] and a sample size of 1,300 participants, a significant difference of 6.5% in VI will be observable post-intervention, using an alpha set at 5%, a beta at 20% and a proportion of discordant pairs of 0.17, i.e. the percentage of participants expected to alter their score in relation to the principal outcome at the post- vs pre-intervention stage.

Analyses were performed under the intention-to-treat principle, i.e. with all participants enrolled in the intervention arm of the pilot PromoVac RCT, with the aim to provide descriptive data for the four study sites. Results were not adjusted for study site baseline criteria. Categorical variables are presented as frequencies (percentages) with a chi-squared Pearson test used for comparisons. Comparative analyses of pre- and post-intervention questionnaires were performed using McNemar’s test for categorical variables and the Wilcoxon signed-rank test for continuous variables. Sensitivity analyses were performed to demonstrate the impact of selected socioeconomic factors on the pre-/post-impact, on the post-/pre-difference of the intervention on VI as well as on VH scores. All statistics were two-tailed. P values of 0.05 or less were considered significant. SAS Institute software version 9.4 (Cary, North Carolina, United States) was used for statistical analyses.

## Results

The PromoVaQ RCT was initially proposed to 4,185 parents between March 2014 and February 2015. Of these, we randomised 2,695 consenting participants from the hospital maternity wards at the four following university hospital centres: the CIUSSS de l’Estrie - CHUS (n = 819), the McGill University Health Centre (n = 627), the CHU Ste-Justine (n = 624) and the CHU de Quebec (n = 625). Participants were equally randomised to the intervention (n = 1,347) or to the control arm (n = 1,348).

**Figure 1:**
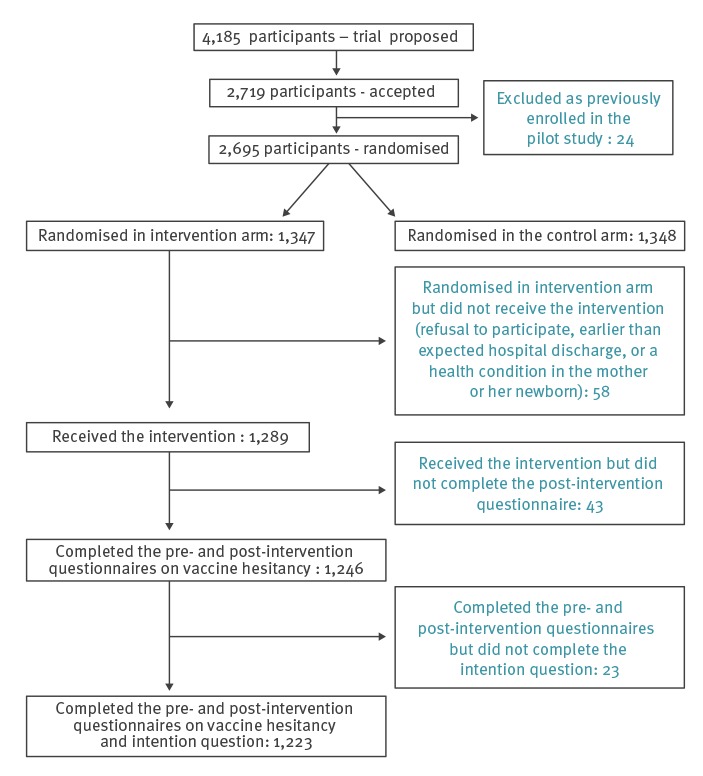
Study flowchart showing the number of participants receiving the intervention, number that completed the pre- and post-intervention questionnaires, Quebec, March 2014–February 2015 (n = 1,223)

In the nested study, we only included the 1,347 participants who had been randomised to the intervention arm; of these, 1,289 received the study intervention. The most frequent reasons not receiving the intervention at this stage (n = 58) were refusal to participate, earlier than expected hospital discharge, or a health condition in the mother or her newborn. Of 1,289 participants who received the intervention, 1,246 completed the pre- and post-intervention questionnaires. Of the latter, 1,223 completed the question on VI pre- and post-intervention (CIUSSS de l’Estrie – CHUS: n = 373; McGill University Health Centre: n = 290; CHU Ste-Justine: n = 265; CHU de Québec: n = 295) and their results are thus the focus of this report; 43 participants completed the pre-intervention questionnaire, received the intervention, but did not complete the post-intervention questionnaire. Compared with the 1,246 participants included in the analyses, these 43 participants were not significantly more vaccine hesitant at the pre-intervention stage (mean Opel scores 27.1 vs 30.3; p = 0.38). However, they were significantly less likely to vaccinate their infant (‘certainly’ category: 78.1% vs 66.7; p = 0.043). Figure 1 depicts the study flowchart.

Table 1 delineates the distribution of participant mothers’ sociodemographic variables by maternity ward. The majority of participants gave birth at term (94.8% at ≥ 37 weeks of pregnancy), nearly half were primigravidas (46.9%), most pregnancies were followed by a gynaecologist-obstetrician (70.4%), and nearly all newborns were healthy, presenting with no condition requiring medical follow-up or assistance (97%). Three quarters of mothers were French speaking (75%) and born in Canada (74.7%). At delivery, a little over half of the mothers were in their 30s (56.7%), held a university degree (54.9%) and were living with a common-law partner (56.4%). Nearly half of participants (48.7%) had an annual family income of at least CAD 80,000 (EUR 54,000). Population characteristics, such as language, age at delivery, educational level, civil status, type of healthcare professional involved in their pregnancy management and annual family income differed significantly between participating maternity wards (all p < 0.05).

**Table 1:** Participants baseline characteristics by maternity ward, Quebec, March 2014–February 2015 (n = 1,223)

Characteristics	Maternity hospital								Total (n = 1,223)	
	CIUSSS de l’Estrie-CHUS (n = 373)		McGill University Health Centre (n = 290)		CHUS Ste-Justine (n = 265)		CHU de Quebec (n = 295)			
	n	%	n	%	n	%	n	%	n	%
**Newborn**										
**Week of delivery**										
< 37	16	4.3	11	3.8	17	6,4	11	3.7	55	4.5
≥ 37	352	94.4	277	95.5	246	92.8	284	96.3	1,159	94.8
Unknown	5	1.3	2	0.7	2	0.8	0	0	9	0.7
**Rank in the family**										
First	179	48.0	131	45.2	128	48.3	135	45.8	573	46.9
Second	126	33.8	110	37.9	87	32.8	116	39.3	439	35.9
Third or more	68	18.2	48	16.6	47	17.7	44	14.9	207	16.9
Unknown	0	0	1	0.3	3	1.1	0	0	4	0.3
**Presence of a disease at birth needing medical follow-up**										
Yes	9	2.4	5	1,7	7	2.6	3	1.0	24	2,0
No	361	96.8	278	95.9	255	96.2	292	99.0	1,186	97,0
Unknown	3	0.8	7	2.4	3	1.1	0	0	13	1.1
**Mother**										
**Language^a^**										
French	343	92.0	110	37.9	200	75.5	264	89.5	917	75.0
English	14	3.8	74	25.5	8	3.0	2	0.7	98	8.0
Both French and English	7	1.9	51	17.6	36	13.6	16	5.4	110	9.0
Other	9	2.4	49	16.9	19	7.2	13	4.4	90	7.4
Unknown	0	0	6	2.1	2	0.8	0	0	8	0.7
**Country of birth^a^**										
Canada	338	90.6	155	53.4	164	61.9	257	87.1	914	74.7
Other	29	7.8	126	43.4	94	35.5	34	11.5	283	23.1
Unknown	6	1.6	9	3.1	7	2.6	4	1.4	26	2.1
**Age at delivery (years)^a^**										
< 20	3	0.8	1	0.3	7	2.6	0	0	11	0.9
20–29	198	53.1	78	26.9	88	33.2	113	38.3	477	39.0
30–39	167	44.8	196	67.6	158	59.6	173	58.6	694	56.7
≥ 40	5	1.3	14	4.8	12	4.5	9	3.1	40	3.3
Unknown	0	0	1	0.3	0	0	0	0	1	0.1
Mean ± SD	29.1 ± 4.7		31.8 ± 4.9		31.1 ± 5.1		30.8 ± 4.6		30.6 ± 4.9	
Median (min-max)	29.0 (18–43)		32.0 (18–50)		32.0 (18–43)		31.0 (20–48)		31.0 (18–50)	
**Education level^a^**										
High school: incomplete	17	4.6	7	2.4	15	5.7	7	2.4	46	3.8
High school: completed	110	29.5	32	11.0	53	20.0	38	12.9	233	19.1
College	95	25.5	50	17.2	41	15.5	67	22.7	253	20.7
University	148	39.7	192	66.2	150	56.6	182	61.7	672	54.9
Unknown	3	0.8	9	3.1	6	2.3	1	0.3	19	1.6
**Civil status^a^**										
Single	17	4.6	16	5.5	18	6.8	9	3.1	60	4.9
Common-law partners	268	71.8	104	35.9	128	48.3	190	64.4	690	56.4
Legally married	84	22.5	161	55.5	111	41.9	95	32.2	451	36.9
Separated or divorced	2	0.5	0	0	4	1.5	0	0	6	0.5
Unknown	2	0.5	9	3.1	4	1.5	1	0.3	16	1.3
**Healthcare professional involved in pregnancy management^a^**										
Family physician	122	32.7	35	12.1	3	1.1	109	36.9	269	22.0
Gynaecologist-obstetrician	213	57.1	237	81.7	257	97.0	154	52.2	861	70.4
Midwife	9	2.4	5	1.7	0	0	2	0.7	16	1.3
None	0	0	1	0.3	0	0	0	0	1	0.1
Both family physician and gynaecologist-obstetrician	20	5.4	3	1.0	3	1.1	30	10.2	56	4.6
Unknown	9	2.4	9	3.1	2	0.8	0	0	20	1.6
**Annual family income^a^**										
< CAD 40,000 (EUR 27,000)	78	20.9	63	21.7	65	24.5	33	11.2	239	19.5
CAD 40,000–79 999 (EUR 27,000-54,000)	133	35.7	81	27.9	72	27.2	61	20.7	347	28.4
≥ CAD 80,000 (EUR 54,000)	159	42.6	125	43.1	117	44.2	194	65.8	595	48.7
Unknown	3	0.8	21	7.2	11	4.2	7	2.4	42	3.4

SD: standard deviation.

^a^ p < 0.05 (Missing value not included). At least one site is different from the other. Statistics present overall differences in socioeconomic factors between maternity wards as a whole. As per the study objectives, no further test was applied to distinguish which site was different from the others.

**Figure 2:**
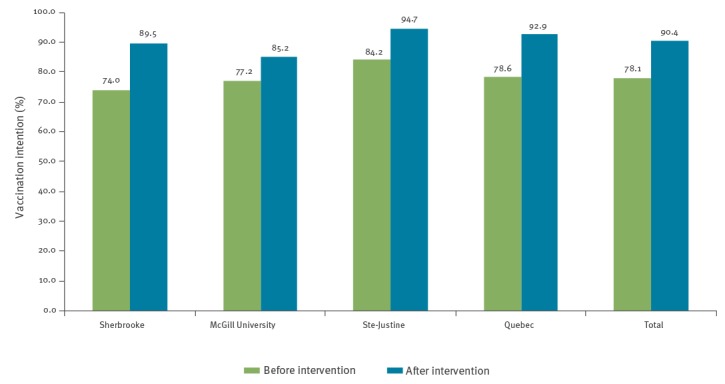
Participants who ‘certainly’ intended to vaccinate their infant at age 2 months before and after the intervention, Quebec, March 2014–February 2015 (n = 1,223)

Figure 2 shows the intention of participants to ‘certainly’ vaccinate their infant at 2 months of age. Prior to the intervention, total intention to ‘certainly’ vaccinate was 78.1% among all participants combined and was significantly different between participating maternity wards (p = 0.02). Following the intervention, the total intention to ‘certainly’ vaccinate rose to 90.4%, a total 12% increase between pre- and post-intervention (p < 0.0001). We found no significant proportion differences (post- vs pre-intervention) between the four study sites (p = 0.24), suggesting that the effect of the intervention was comparable at each site. A significant rise in intention to ‘certainly’ vaccinate was observed at each site post-intervention (p < 0.0001 each site). The very small number of participants in the ‘certainly not’ category of vaccination intention makes it difficult to accurately measure the effect of the study intervention; we observed a shift from 0.7 to 0.2% in the ‘certainly not’ category (Table 2).

**Table 2:** Intention of participants to vaccinate their infant at age 2 months before and after the intervention, Quebec, March 2014–February 2015 (n = 1,223)

Intention to vaccinate	Maternity hospital								Total (n = 1,223)	
	CIUSSS de l’Estrie-CHUS (n = 373)		McGill University Health Centre (n = 290)		CHUS Ste-Justine (n = 265)		CHU de Quebec (n = 295)			
	n	%	n	%	n	%	n	%	n	%
**Pre-intervention**										
Certainly not	4	1.1	3	1.0	1	0.4	0	0.0	8	0.7
Probably not	4	1.1	8	2.8	4	1.5	2	0.7	18	1.5
Probably	89	23.9	55	19.0	37	14.0	61	20.7	242	19.8
Certainly**^a^**	276	74.0	224	77.2	223	84.2	232	78.6	955	78.1
**Post-intervention**										
Certainly not	1	0.3	1	0.3	1	0.4	0	0.0	3	0.2
Probably not	1	0.3	2	0.7	0	0.0	0	0.0	3	0.2
Probably	37	9.9	40	13.8	13	4.9	21	7.1	111	9.1
Certainly**^a^**	334	89.5	247	85.2	251	94.7	274	92.9	1,106	90.4

**^a^** p value: Vaccination intention post- vs pre-intervention (< 0.0001).

Participant VH significantly decreased post-intervention. Overall, the combined data from the four study sites showed that the relative proportion of participants with lowest VH (score 0–29) rose from 55.9% to 78.8% (41% increase), while those with intermediate and highest levels of VH (score 30–49 and > 50) decreased from 44.1% to 21.1% (Table 3). Prior to the intervention, 15.6% of our overall population displayed high VH (> 50%). This fraction decreased to only 5.2% post-intervention (p < 0.0001). The mean Opel score significantly decreased at each site between pre- and post-intervention evaluations (p < 0.0001): -12.1% (IC95: -13.6%; -10.6% - CIUSSS de l’Estrie - CHUS), -8.0% (-9.4%; -6.5% - McGill University Health Centre), -10.8% (-12.9%; -9.1% - CHU Ste-Justine) and -11.5% (-13.1%; -9.9% - CHU de Québec). Overall, the mean Opel score went from 27.1% to 16.4%, for a 40% reduction in VH (p < 0.0001) (Table 3).

**Table 3:** Hesitation of participants to vaccinate their infant at age 2 months before and after the intervention, Quebec, March 2014–February 2015 (n = 1,223)

Hesitation to vaccinate^a^	CIUSSS de l’Estrie-CHUS (n = 373)		McGill University Health Centre (n = 290)		CHUS Ste-Justine (n = 265)		CHU de Quebec (n = 295)		Total (n = 1,223)	
	n	%	n	%	n	%	n	%	n	%
**Pre-intervention**										
0–29	201	53.9	159	55.2	138	52.5	184	62.4	682	55.9
30–49	105	28.2	72	25.0	92	35.0	78	26.4	347	28.5
≥ 50	67	18.0	57	19.8	33	12.5	33	11.2	190	15.6
Mean Opel Score	28.2		28.7		27.3		24.0		27.1	
**Post-intervention**										
0–29	296	79.4	207	71.4	202	76.2	259	87.8	964	78.8
30–49	59	15.8	60	20.7	51	19.2	25	8.5	195	15.9
≥ 50	18	4.8	23	7.9	12	4.5	11	3.7	64	5.2
Mean Opel Score	16.1**^b^**		20.7^b^		16.5**^b^**		12.5**^b^**		16.4**^b^**	

VH: vaccine hesitant.

^a ^Categories were defined as follows: Opel score: 0–29 = low level VH; 30–49 = moderate level VH; 50 and higher = high level VH.

^b^ p value: mean Opel score post- vs pre-intervention (< 0.0001).

Table 4 shows the results from a sensitivity analysis conducted to determine if there were any differences in VI and VH, when socioeconomic or cultural characteristics that were found to be different between the sites in Table 1 were analysed. The results supported the finding that the pre-/post-impact of the intervention on both VI and VH scores was effective, irrespective of the differing characteristics (Table 4). An exception was that the pre-/post-impact of the intervention was not effective when a midwife was in charge of pregnancy management. This result should, however, be interpreted with caution as only 16 participants were in that category.

**Table 4:** Intention and hesitation of participants to vaccinate their infant at 2 months before and after the intervention, by mothers’ characteristics, Quebec, March 2014–February 2015 (n = 1,223)

Participant characteristics	VI at age 2 months					VH at age 2 months									
	Total	Pre intervention	Post intervention	Pre-post	Diff- in-diff (interaction)	Pre-intervention				Post-intervention				Pre-post	Diff- in-diff (interaction)
	n	%	%	p value	p value	0–29%	30–49%	≥ 50%	Mean Opel Score	0–29%	30–49%	≥ 50%	Mean Opel Score	p value^a^	p value^a^
**Language**															
French	917	77.8	91.2	< 0.0001	0.0621	58.45	27.59	13.96	26.0	82.55	13.20	4.25	14.7	< 0.0001	0.0136
English or other	298	78.9	87.9	< 0.0001		47.65	31.21	20.13	30.4	67.45	24.16	8.39	21.8	< 0.0001	
**Country of birth**															
Canada	914	77.8	91	< 0.0001	0.0914	60.50	25.93	13.57	25.3	84.25	11.60	4.16	13.9	< 0.0001	0.0032
Other	283	79.1	88.3	< 0.0001		41.70	36.40	20.85	32.6	61.84	29.68	8.48	24.4	< 0.0001	
**Age at delivery (years)**															
< 30	488	74.4	90.8	< 0.0001	0.0029	52.87	31.56	15.57	28.4	78.48	17.01	4.51	16.4	< 0.0001	0.0124
≥ 30	734	80.5	90.2	< 0.0001		57.77	26.29	15.53	26.3	79.02	15.26	5.72	16.5	< 0.0001	
**Educational level**															
High school: incomplete /completed	279	71.3	92.8	< 0.0001	0.0003	42.29	37.63	19.35	32.2	72.76	19.71	7.53	19.2	< 0.0001	< 0.0001
College	253	77.9	88.5	< 0.0001		54.15	27.27	18.58	29.1	79.84	17.00	3.16	15.2	< 0.0001	
University	672	80.8	90	< 0.0001		62.65	24.40	12.80	24.1	81.55	13.39	5.06	15.5	< 0.0001	
**Living with a partner**															
Yes	1,141	78.1	90.1	0.0017	0.2148	56.70	28.31	14.81	26.7	79.49	15.43	5.08	16.1	< 0.0001	0.2302
No	66	74.2	93.9	< 0.0001		39.39	31.82	27.27	34.7	66.67	24.24	9.09	22.4	< 0.0001	
**Healthcare professional involved in pregnancy management**															
Family physician	1,186	78.4	90.9	< 0.0001	0.5935	55.99	28.75	15.01	26.9	79.34	15.68	4.97	16.2	< 0.0001	0.3430
Midwife	16	56.3	56.3	1.0000		43.75	18.75	37.50	33.5	56.25	25.00	18.75	26.1	0.0039	
**Annual family income**															
< CAD 40,000 (EUR 27,000)	239	77.4	89.1	< 0.0001	0.8711	44.35	35.15	19.67	32.2	66.53	25.10	8.37	22.2	< 0.0001	0.0003
CAD 40,000–79,999 (EUR 27,000–54,000)	347	74.9	87.9	< 0.0001		47.84	33.43	18.73	30.4	76.66	17.58	5.76	17.1	< 0.0001	
≥ CAD 80,000 (EUR 54,000)	595	80.5	92.9	< 0.0001		65.71	22.52	11.76	22.9	85.71	11.09	3.19	13.4	< 0.0001	
**Child's rank in the family**															
First	573	67.2	86.7	< 0.0001	< 0.0001	50.6	31.24	18	29.5	78.4	17.1	4.5	16.8	< 0.0001	< 0.0001
Second	439	88.8	94.5	< 0.0001		61.1	26.2	12.5	25.1	79.7	14.1	6.2	16.3	< 0.0001	
Third or more	207	85.5	91.8	0.0029		59.4	25.6	14	24.5	78.7	15.9	5.3	15.3	< 0.0001	
**Number of parents**															
Both^b^	866	76.9	89.5	< 0.0001	0.3391	57.8	27.4	14.6	26.5	82	13.2	4.8	15.2	< 0.0001	0.051
Mother only	331	81.9	92.7	< 0.0001		51.4	20.2	17.8	28.3	71.6	22.7	5.7	18.9	< 0.0001	

Diff-in-Diff: Difference-in-Difference; VH: vaccine hesitant; VI: vaccination intention.

^a^ For Mean Opel Score.

^b^ Includes heterosexual and same sex couples.

With regard to the intention to vaccinate their infant at 2 months of age, results from sensitivity analyses demonstrated that the mother’s age at delivery, i.e. being under or 30 years old, less educated, i.e. only completed high school, or being a primipara, all significantly increased the difference in pre-/post-impact of the intervention between categories. We also found that the VH scores were significantly lower in mothers who were French speakers, of Canadian origin, aged 30 years or younger, had completed at least high school, were in the middle-class income category (CAD 40,000–79,000/EUR 27,000–54,000) and primipara.

## Discussion and conclusions

This study assessed the impact of an MI-based intervention conducted with parents post-partum regarding VH and VI for their newborn. We found that the pre-/post-impact of the intervention was effective, irrespective of the potential confounding sociodemographic and cultural factors. These results highlight the generalisability of this novel approach to help parental decision-making regarding immunisation and reduce VH.

A systematic review of literature on currently available interventions aimed at reducing parental vaccine refusal and hesitancy, concluded that reports on such interventions were scarce and given the lack of data to adequately inform policy and decision makers well-designed trials were needed [25]; the results of our study contribute to partially fill this knowledge gap. Our results, showing that a tailored MI-based intervention can raise parental VI, are supported by the conclusions of a 2018 Cochrane database systematic review and meta-analysis [9]. They included seven RCTs and three cluster-RCTs, covering a total of 4,527 participants. Although the studies were at risk of bias and therefore had a low-certainty of evidence, the overall conclusion was that face-to-face interventions can slightly improve VI compared with standard care (standardised mean difference 0.55; 95% CI: 0.24–0.85) [9]. Our PromoVac strategy is a patient-centred approach aimed at increasing parental motivation through exploring and solving personal inherent ambivalences towards immunisation of their infant. While some face-to-face interventions have proven more effective in populations for whom immunisation knowledge was a barrier rather than VI per se [9], our strategy was effective in participants with a high degree of VH pre-intervention. Indeed, parents who fell into the ‘probably’ category for VI, i.e. those who were most likely to be vaccine-hesitant, were those whose VI shifted the most post-intervention. Overall, 46% of participants in the ‘probably’ category for VI transitioned to a more favourable position, i.e. in the ‘certainly’ category (data not shown).

Our results indicate that an MI-based intervention is effective in parents presenting high levels of VH – the population that has been identified as crucial for effective intervention; Leask et al. emphasised that these parents’ needs must be met in order for them to be able to modify their perception of childhood vaccination [26]. We found that the MI-based intervention matched participant’s expectations and needs and we believe this was attributable to the MI approach and techniques used in our intervention. For example, we facilitated a highly respectful and empathetic discussion of participants’ concerns about childhood vaccination, which in turn, contributed to help build a trusting relationship between parents and research assistants. In addition, we ensured parents were given an opportunity to freely voice their concerns and questions about immunisation in the absence of any judgmental attitude from the healthcare professional. We believe that this is the distinctive feature of our intervention and may, in part, explain the positive results. A Cochrane review led by Kaufman et al. concluded that a face-to-face intervention may not impact positively vaccine coverage when strictly based on providing practical and logistical information regarding vaccination without any consideration for the parents’ beliefs on the matter [9]. Results from an RCT that enrolled adolescents to assess the impact of MI on human papillomavirus vaccination [27] support the approach we choose among available options. Furthermore, our approach is in line with a 2017 Cochrane review suggesting that parents expect to be provided balanced information, as to the risks and advantages of immunisation, in a simple manner by a professional they trust. When these conditions are not met, uptake of vaccination may decrease [28]. Our study intervention was adapted to each parent’s individual needs, which avoided the backfire that providing unnecessary or unsolicited advice can exert [29]. Also in support for our MI-based intervention is its efficacy in spite of sociodemographic factors. Indeed, it seemed to be more effective, i.e. it exerted a greater difference post-intervention with regard to intention to treat, whether mothers were aged 30 years or younger, had completed no more than high school education, or were primipara. In fact, despite their even lower pre-intervention scores, these mothers had post-intervention scores that were comparable to those of the older, more educated and experienced mothers.

Our results demonstrated the MI-based intervention consolidated decision making of participants who were immunisation favourable at baseline. Post-intervention, an additional 41% fell into the 0–30 Opel score category (lowest VH) and an additional 12% into the ‘certainly’ category of VI. Interestingly, as reported in a meta-analysis, VI may be predictive of behaviour [30], suggesting that parents’ intention may be translated into action to vaccinate their child. Several studies have shown that VI is correlated with the decision and behaviour to vaccinate [31,32]. One study on vaccination against influenza in Dutch healthcare personnel demonstrated that VI was a significant predictor of vaccination behaviour with an odds ratio of 15.50 (95% CI: 9.24–25.99) [33].

### Strengths and limitations

This study builds on a variety of strengths increasing external validity including, (i) a unique parent-centred MI-based intervention, (ii) a parent-tailored approach, (iii) the use of validated reliable questionnaires and tools to secure internal validity and outcome assessment (e.g. use of validated questionnaires, standardised research assistant training between sites, use of a standard operating procedures manual, a trial period (refer to the study protocol [17])), and (iv) a considerably large number of participants enrolled at four university hospital centres across the Province of Quebec. The study intervention was standardised and thus reproducible in other maternity wards as indicated by the consistent results across all maternity wards and there being no significant differences for the main outcome. The results are also generalisable to the province, as the study was built upon a large and representative sample from four university hospital maternity wards (accounting for over 20% of all births) in the Province of Quebec and included both English and French speakers. The study population was diverse and suited to our intention to increase the validity of our results. In addition, Quebec’s Provincial Health Insurance Plan covers the hospitalisation of mothers for childbirth, so financial considerations do not affect the decision whether to deliver at a hospital maternity ward or at home. Our results also demonstrate that although the different study-site populations were heterogeneous, as shown by their baseline characteristics, the study intervention had the same impact on participants despite regional population disparities.

This study has some limitations. For instance, the initial reason for the refusal to participate was not collated despite the fact that it might have enriched our understanding of the enrolled population and potential biases. Also, mothers who gave birth at home or in birthing centres were not included in the study and they may have had different opinions regarding childhood vaccination, such as a higher tendency not have their children immunised as midwife-assisted birth (performed at home or in birth centres in Quebec) was associated with an incomplete immunisation status in Quebec and Canada [4,34-36]. However, these women only represent less than 3% of all births in the Province of Quebec [37], thus even if these women would have been approached to participate, we believe study results would not have changed in a predominant way. An additional concern is that this is an RCT-nested study, so participants in the RCT who were randomised to the standard-of-care arm did not complete the VH question or the questionnaire on VH at hospital discharge. Only baseline VI and VH were recorded for these participants. Our study results are thus mitigated by this limitation. In addition, our conclusions lack some degree of validity, as we were unable to assess whether the Hawthorne effect may have contributed to the participants’ VI and VH. The Hawthorne effect is described as being a bias related to a change in behaviour of participants/staff following their recognition of being observed or through desirability concerns, which can alter results [38]. As this was a parallel, rather than a cluster RCT, staff and patients were well aware that of the study intervention, which may have influenced practice or beliefs in the study setting. Another limitation is that external generalisability may be compromised by the fact that this study was conducted in tertiary care centres. Patients giving birth in primary care centres, which represent 75% of all births in the Province of Quebec [39], may have other opinions or may have received the study intervention differently. However, vaccine coverage of children born in areas with and without tertiary care centres are similar throughout Quebec [4], which reduces the effect of this bias. Moreover, the fact that the post-intervention questionnaire was administered to participants immediately following the study intervention may have positively influenced their answers and VI, as per social desirability bias. However, this methodological approach was adopted in order to measure the direct effect of the study intervention and not be mitigated by other external factors on a more long-term basis.

### Conclusion

To our knowledge, this is the first study of its kind comparing the efficacy of an MI-based intervention on VI and VH in a large number of participants pre- and post-intervention. Although non-controlled (as per the study’s design), our results show the efficacy of our MI-based post-partum intervention in providing parents of newborns with individually-tailored immunisation decision-making and educational support. This intervention reduced parental VH while enhancing VI for their infant at 2 months of age. Going forward, we aim to assess the impact of such an intervention on child vaccine coverage at later ages and to correlate these with VI and VH scores.

## Supplementary Data

218000 Supplement S1
