# Supplementary material for: Promoting vaccination in maternity wards ─ motivational interview technique reduces hesitancy and enhances intention to vaccinate, results from a multicentre non-controlled pre- and post-intervention RCT-nested study, Quebec, March 2014 to February 2015
Source: Euro Surveill. 2019 Sep 5;24(36):1800641. doi: 10.2807/1560-7917.ES.2019.24.36.1800641 (PMC6737828; doi:10.2807/1560-7917.ES.2019.24.36.1800641)
Supplement: Supplement S1 [file 1800641_GAGNEUR_SupplementS1.pdf]

Appendix - Quebec immunization schedule in 2014-2015

Vaccines	Recommended age of administration (months)				
	2	4	6	12	18
DTaP-Polio-Hib	X	X	X		X
Pneumococcal conjugate	X	X		X	
Rotavirus	X	X			
Meningococcus				X	
MMR-Varicella				X	
MMR					X

This supplementary material is hosted by Eurosurveillance as supporting information alongside the article “Promoting vaccination in maternity wards – motivational interview technique reduces hesitancy and enhances intention to vaccinate, results from a multicentre non-controlled pre- and post-intervention RCT-nested study, Quebec, March 2014 to February 2015” on behalf of the authors who remain responsible for the accuracy and appropriateness of the content. The same standards for ethics, copyright, attributions and permissions as for the article apply. Supplements are not edited by Eurosurveillance and the journal is not responsible for the maintenance of any links or email addresses provided therein.
